# Different methylation signatures at diagnosis in patients with high-risk myelodysplastic syndromes and secondary acute myeloid leukemia predict azacitidine response and longer survival

**DOI:** 10.1186/s13148-021-01002-y

**Published:** 2021-01-14

**Authors:** M. Cabezón, R. Malinverni, J. Bargay, B. Xicoy, S. Marcé, A. Garrido, M. Tormo, L. Arenillas, R. Coll, J. Borras, M. J. Jiménez, M. Hoyos, D. Valcárcel, L. Escoda, F. Vall-Llovera, A. Garcia, L. L. Font, E. Rámila, M. Buschbeck, L. Zamora, CETLAM group

**Affiliations:** 1 Hematology Laboratory Service, ICO Badalona-Hospital Germans Trias I Pujol, Myeloid Neoplasms Group, Josep Carreras Leukemia Research Institute (IJC), Badalona, Spain; 2 Departament de Medicina, Universitat Autònoma de Barcelona, Badalona, Spain; 3 Cancer and Leukemia Epigenetics and Biology Program, Josep Carreras Leukemia Research Institute (IJC), Campus ICO-GTP-UAB, Badalona, Spain; 4 Hematology Service, Hospital Son Llàtzer, Palma de Mallorca, Spain; 5 Hematology Service, Hospital de Sant Pau, Barcelona, Spain; 6 Hematology Service, Hospital Clínico de Valencia, Valencia, Spain; 7 Hematology Service, Hospital del Mar, Barcelona, Spain; 8 Hematology Service, ICO Girona - Hospital Josep Trueta, Girona, Spain; 9 Hematology Service, Hospital Vall D’Hebron, Barcelona, Spain; 10 Hematology Service, Hospital Joan XXIII, Tarragona, Spain; 11 Hematology Service, Hospital Mútua de Terrassa, Terrassa, Spain; 12 Hematology Service, Hospital Arnau de Vilanova, Lleida, Spain; 13 Hematology Service, Hospital Verge de La Cinta, Tortosa, Spain; 14 Hematology Service, Hospital Parc Taulí, Sabadell, Spain; 15 Program for Predictive and Personalized Medicine of Cancer, Germans Trias I Pujol Research Institute (PMPPC-IGTP), Badalona, Spain

**Keywords:** Myelodysplastic syndromes, Secondary acute myeloid leukemia, DNA methylation, Hypomethylating agents, Epigenetic drugs, Prognostic factors, Azacitidine

## Abstract

**Background:**

Epigenetic therapy, using hypomethylating agents (HMA), is known to be effective in the treatment of high-risk myelodysplastic syndromes (MDS) and acute myeloid leukemia (AML) patients who are not suitable for intensive chemotherapy and/or allogeneic stem cell transplantation. However, response rates to HMA are low and there is an unmet need in finding prognostic and predictive biomarkers of treatment response and overall survival. We performed global methylation analysis of 75 patients with high-risk MDS and secondary AML who were included in CETLAM SMD-09 protocol, in which patients received HMA or intensive treatment according to age, comorbidities and cytogenetic.

**Results:**

Unsupervised analysis of global methylation pattern at diagnosis did not allow patients to be differentiated according to the cytological subtype, cytogenetic groups, treatment response or patient outcome. However, after a supervised analysis we found a methylation signature defined by 200 probes, which allowed differentiating between patients responding and non-responding to azacitidine (AZA) treatment and a different methylation pattern also defined by 200 probes that allowed to differentiate patients according to their survival. On studying follow-up samples, we confirmed that AZA decreases global DNA methylation, but in our cohort the degree of methylation decrease did not correlate with the type of response. The methylation signature detected at diagnosis was not useful in treated samples to distinguish patients who were going to relapse or progress.

**Conclusions:**

Our findings suggest that in a subset of specific CpGs, altered DNA methylation patterns at diagnosis may be useful as a biomarker for predicting AZA response and survival.

## Background

The term epigenetics refers to all the information transmitted through cell division that is not encoded in the DNA sequence. The methylation of DNA, encodes some epigenetic information related to the transcriptional state of genes [1]. DNA methylation is catalyzed by DNA methyltransferases (DNMTs) that transfer a methyl group to a cytosine residue (5-methylcytosine) within a CpG dinucleotide. The large majority of CpGs in the genome are methylated and contribute to stable repression of repeated sequences in heterochromatin stability [2]. Regions enriched in CpG, called CpG islands, are also found in the promoters of genes. The majority of these CpG islands are unmethylated in cells of normal tissues regardless of their differentiation state [1]. This lack of methylation in promoter-associated CpG islands allows gene expression, if the appropriate transcription factors are present and the chromatin structure allows access to them [3, 4]. Many human diseases, and in particular cancers, are associated with aberrations in the DNA methylation profile [1, 5–7]. Neoplastic cells frequently display global DNA hypomethylation with localized hypermethylation of CpG islands [4]. Hypomethylation may play a role in carcinogenesis through activation of oncogenes [8] and chromosomal instability [9]. In contrast, aberrant hypermethylation of CpG islands in cancer is clearly associated with gene silencing and contributes to the inactivation of tumor suppressor genes [4, 10, 11]. Baylin and Jones estimated that the inactivation of tumor suppressor genes by gaining DNA methylation occurs as often as their inactivation by mutations [7, 12]. In contrast to genetic alterations, epigenetic changes are potentially reversible by pharmacological inhibition of DNA methylation and histone deacetylation [13], thus providing a potential point for therapeutic intervention.

The azanucleosides, azacitidine (AZA) and decitabine, are incorporated as analog of cytidine into DNA and inhibit DNA methyltransferase through covalent binding [14]. As a consequence of this function azanucleosides are commonly referred to as hypomethylating agents (HMAs).

The reactivation of abnormally silenced tumor suppressor genes, DNA repair genes, and microRNAs was long thought to be the main mechanism by which HMA exerts anti-tumor activity [15–17]. However, more recently, HMA have shown to up regulate endogenous retrovirus transcripts, which form cytoplasmic double-stranded RNA and trigger autonomous and immune-cell induced cell death [18–20]. In addition, azanucleosides induce cytotoxic effects by impacting on DNA and, in the case of AZA also RNA, metabolism [21, 22].

Myelodysplastic syndromes (MDS) refer to a heterogeneous group of aging-related myeloid neoplasms originating in hematopoietic stem and progenitor cells. MDS are characterized by ineffective hematopoiesis and increased risk of progression to secondary acute myeloid leukemia (sAML). Genetic and epigenetic changes contribute to the pathogenesis of the diseases [15, 23–27]. In the context of epigenetics, altered DNA methylation patterns have been observed in MDS. In particular, the hypermethylation of CpG islands and the silencing of linked genes appear in early stages of the disease and are associated with poor prognosis and disease progression [28–30]. Azacitidine has been shown to prolong overall survival compared with conventional care regimens and thus, it is recommended as first-line treatment for most patients with higher-risk MDS which are not eligible for allogeneic stem cell transplantation [14, 31]. Half of the patients do not respond favorably to the treatment and virtually all responders eventually relapse [32]. A major limitation for the clinical management is that several months of treatment are required before the efficacy of the therapy can be assessed. At present, we lack a robust biomarker for predicting treatment response. In particular, there is debate about the informative potential of DNA methylation at diagnosis. Some studies have shown an association between pretreatment DNA methylation profiles and response to HMA [33, 34], while others have not [35–37].

While HMAs are undoubtedly helpful in the treatment of MDS patients, there is an urgent need to find prognostic and response-predictive biomarkers that could determine whether a patient will respond to HMAs. This would avoid progression under treatment, the delay of other therapeutical options and the development of unwanted side effects. To address this need, the aim of our study was to assess DNA methylation patterns at diagnosis and follow-up in patients with high-risk MDS and sAML and determine if there is a methylation profile that distinguishes patients according to treatment response and overall survival (OS).

## Results

### Patient and sample characteristics

This study included a total of 75 patients with high risk MDS or sAML. The main clinical and hematological characteristics of these patients at diagnosis are described in Table 1.

**Table 1:** Main clinical and hematological characteristics of patients at diagnosis (*n* =75)

Variable	Median (range)	*N* (%) (*n *= 75)
Age, years	66 (32–83)	
< 65		34
≥ 65		41
Gender		
Male		52 (69)
Female		23 (31)
Hemoglobin level, g/L	91 (57–136)	
< 100		50
≥ 100		25
Leukocyte count, × 10^9^/L	2.9 (0.8–108)	
< 4		47
> 4 and < 11		14
≥ 11		14
Platelet count, × 10^9^/L	68 (5–536)	
< 100		48
≥ 100		27
Neutrophil count, × 10^9^/L	1.23 (0.09–13.55)	
< 0.8		27
≥ 0.8		47
No data		1
Blasts in PB, %	0 (0–74)	
< 5		55
≥ 5		20
Blasts in BM, %	15 (0–92)	
< 20		49
≥ 20		26
Cytogenetic		
Normal karyotype		28
Abnormal karyotype		45
Uninformative CC		2
Cytogenetic category (IPSS-R)		
Very good		1
Good		30
Intermediate		20
Poor		6
Very poor		16
Uninformative CC		2
IPSS risk group (only MDS patients: 49)		
Intermediate-1		6
Intermediate-2		23
High		19
No data		1
IPSS-R risk group (only MDS patients: 49)		
Low		1
Intermediate		9
High		16
Very high		21
No data		2
Treatment		
AZA	38	
Other treatment (ICE plus AZA and/or ASCT)	37	

*PB* peripheral blood, *BM* bone marrow, *Uninformative CC* cases with no metaphases, *IPSS* International Prognostic Score System, *IPSS-R* Revised IPSS, *WHO* World Health Organization, *AZA* azacitidine, *ICE* idarubicin, cytarabine and etoposide, *ASCT* allogeneic stem cell transplantation

We analyzed 156 bone marrow aspirates that were collected at diagnosis and during follow-up. Of these samples, 108 were from high-risk MDS and 48 from sAML patients. The number of samples at diagnosis and follow-up are shown in Additional file 1: Table 1. In two patients follow-up samples were available, but a sample at diagnosis was lacking. Approximately half of all the patients received AZA treatment, while the other half underwent intensive chemotherapy followed or not by ASCT. Treatment response to AZA was assessed following the criteria defined by the 2006 IWG for MDS patients [38]. Based on these criteria, our study included 21 responders and 16 non responders to AZA treatment. The median follow-up of the patients was 12 months, ranging from 0 to 72 months. Additional file 1: Table 2 summarizes the number of patients according to the response to all treatments.

The median (OS) of the whole series (75 patients) was 12.6 months (95% CI 8.4, 16.7), but analysing it according to treatment type, we observed that median OS in patients that received AZA (*n* =38) and other treatment (*n* =37) was 12 months (range 7.8–16.2) and 12.6 months (range 3–22.1), respectively. Regarding progression free survival (PFS), for the entire cohort was 10.4 months (95% CI 8.8, 11.9), and analysing by treatment type, the median PFS for patients that received AZA was 10.4 months (range 8.3–12.4), whereas it was 9.5 months (range 6.4–12.6) for patients that received other treatment (Additional file 1: Figure 1). No significant differences in survival were seen between patients who received AZA and those who received other treatment.

### Global genome-wide methylation analysis of bone marrow distinguishes healthy controls from the heterogeneous group of high risk MDS and sAML patients

**Fig. 1:**
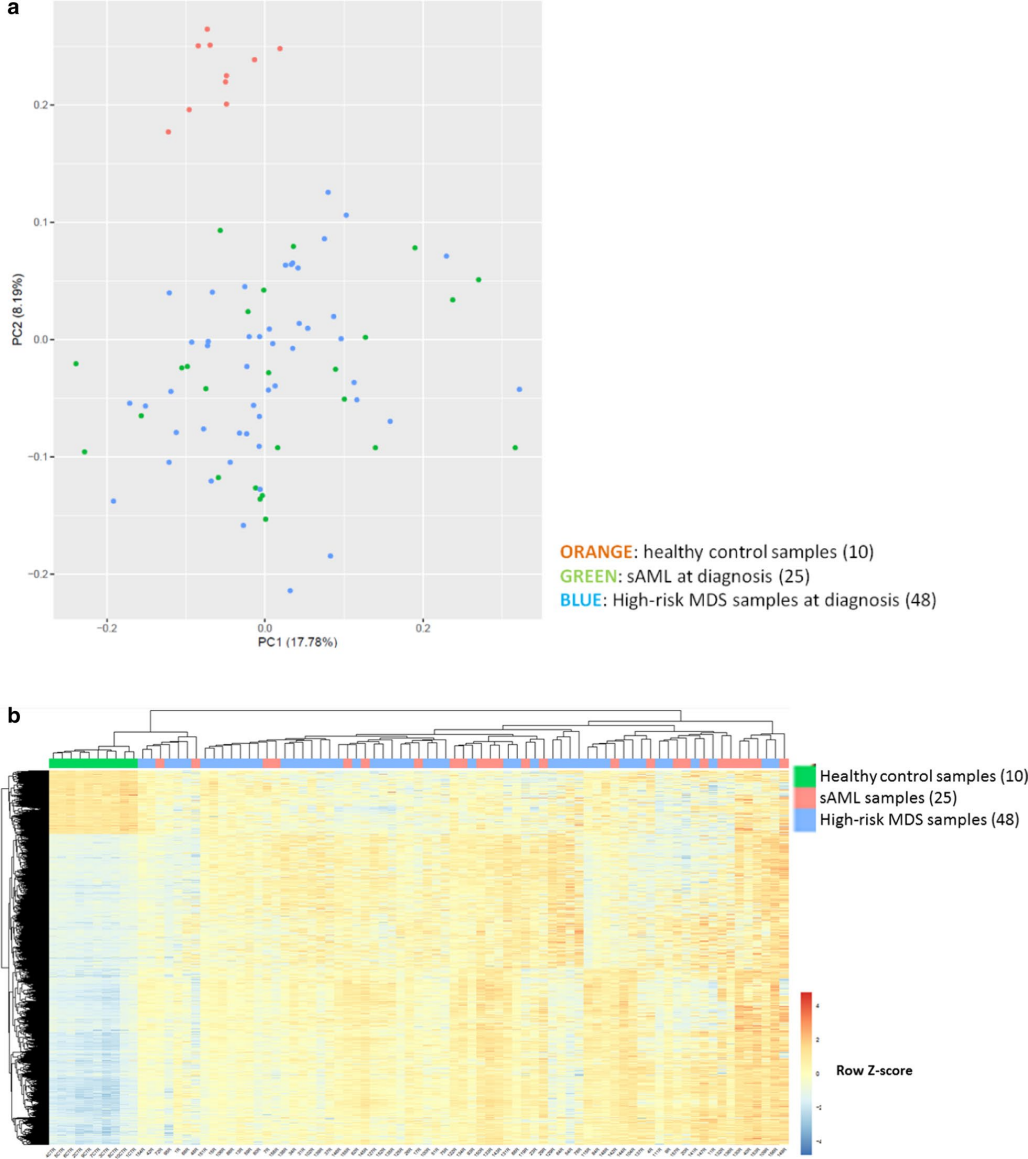
DNA methylation profiles distinguish patients and healthy controls. **a** Principal component analysis shows a separation between patient and control samples after unsupervised analysis. We have analyzed a total of 25 bone marrow samples taken at diagnosis from patients with secondary acute myeloid leukemia (sAML) and 48 from patients with myelodysplastic syndrome (MDS). **b** Heatmap showing major methylation in neoplastic samples compared to healthy controls

Bone marrow aspirates were routinely collected during the MDS and sAML diagnostic process. Here, we analyzed global genome-wide methylation profile from 156 samples from patients and ten hematopoietic stem cell donors as healthy controls. Given that the controls were related donors their median aged (50 years; range 28–63 years) were younger than patients age (median: 66 years; range 32–83 years). Specifically, we determined the methylation level of 450,000 CpG sites using the Illumina Infinium array platform. We analyzed samples in different batches and included the same two reference samples to control for potential batch effects. Importantly, the methylation profiles of these two samples were always the same, and all samples fulfilled the trial quality control criteria. Unsupervised analysis of diagnostic samples by principal component analysis segregated neoplastic samples from healthy control samples, showing homogeneity in the controls and the heterogeneity of the neoplastic samples at diagnosis (Fig. 1a). High-risk MDS and sAML samples did not separate but were intermixed, reflecting the relationship of the two diseases and their intrinsic heterogeneity (Fig. 1a). Furthermore, we did not observe segregation according to cytogenetic group or treatment response at this global level of analysis (data not shown).

To identify changes in the DNA methylation profile associated with disease, we compared the DNA methylation patterns from high risk MDS and sAML patients at diagnosis (*n* =73; 48 MDS and 25 sAML) with those from healthy controls (*n* =10). Statistical analysis identified 40,395 probes that indicated significantly differentially methylated sites between the two groups (*P* < 0.05). To be more restrictive, we focused on sites that had an absolute mean differential methylation between the two groups greater than 0.2, with 0 being un-methylated and 1 being fully methylated (> 0.2 methylation difference, *P* < 0.05). Up to 8,247 probes met these criteria. Of these, 6,841 probes were more methylated in patient samples than in healthy controls, and 1,406 probes were less methylated in patients (Fig. 1b). To better understand the differences between patients and healthy controls, we focused our studies on probes that were located in the promoter region, defined from − 2000 to + 500 bp from the transcription start site (TSS). Following the trend of all differentially methylated sites, hypermethylated sites in gene promoters were more frequent than hypomethylated. Specifically, 1213 gene promoters had at least one CpG site more methylated in MDS and sAML patients than in healthy controls, while 352 gene promoters showed the opposite. Focusing on the predominant hypermethylation of neoplastic samples, we found that in 109 genes at least four sites were more methylated in their promoter region. Gene Ontology (GO) of pathways analysis indicated an enrichment of genes related to cadherin and Wnt signaling pathways (for more information see Additional file 1: Figure 2). Taken together, these results further substantiate previous studies showing that DNA methylation profiles are altered in high-risk MDS and sAML samples.

### A methylation signature at diagnosis predicts AZA response

**Fig. 2:**
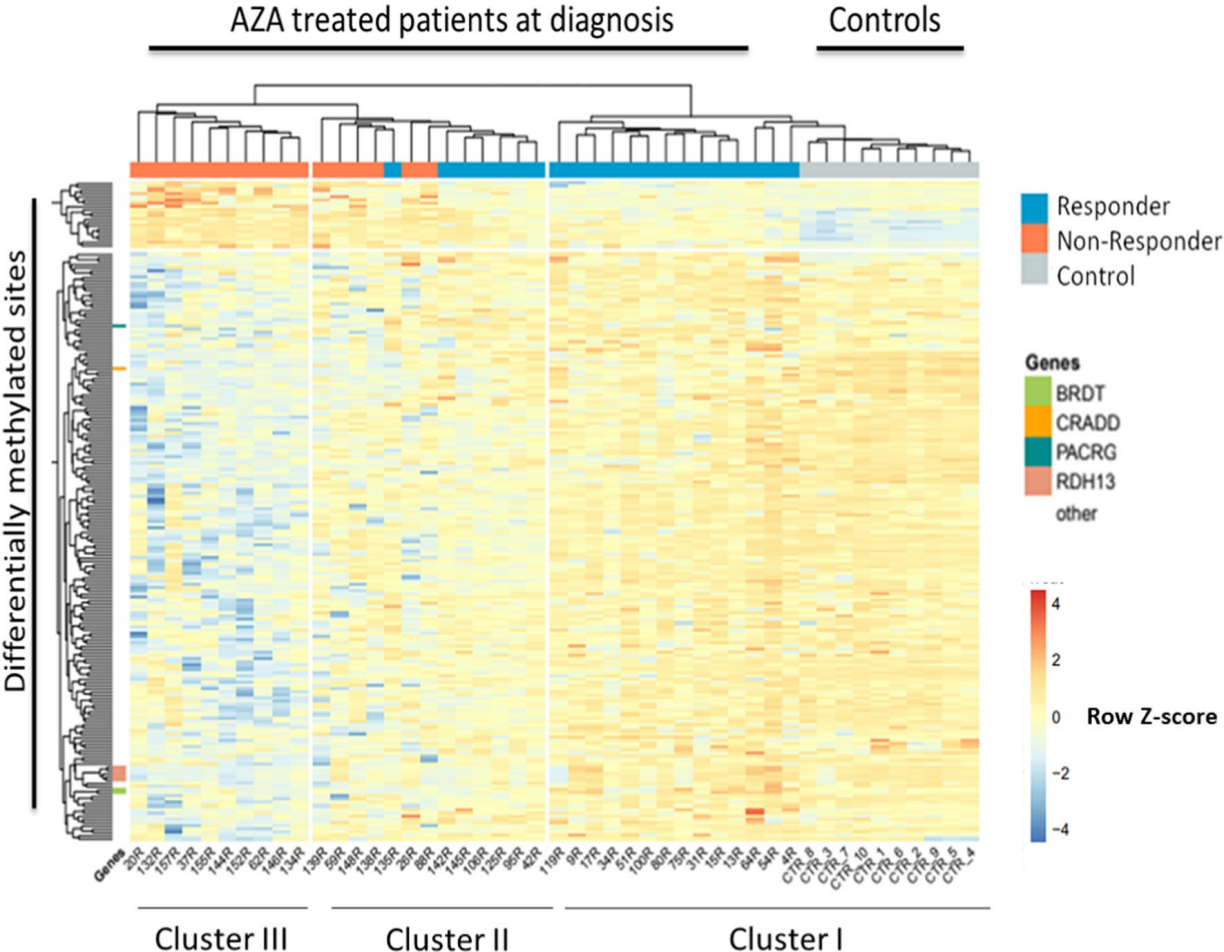
Methylation profile of 200 probes at diagnosis allow distinguishing responders and non-responders to azacitidine (AZA) treatment. Heatmap showing the hierarchical clusterization of the 200 probes obtained with combined rank analysis that are differentially methylated between responders and non-responders in bone marrow samples at diagnosis. Selected genes associated with differentially methylated CpGs are indicated

Taking into account the partial response of patients to AZA, response-predicting biomarkers are urgently needed. In order to identify a potential biomarker, we focused on the 37 patients treated with AZA in our cohort from whom a sample was available at diagnosis. Of these patients, 21 favorably responded to the treatment, and 16 were resistant and did not respond. Comparing the two groups, we identified a methylation signature, defined by 200 probes that allowed differentiation. When we performed hierarchical clustering with the values of these 200 probes, we classified patients into two major clusters (Fig. 2). Cluster I included exclusively responders plus control samples. The second large cluster was further subdivided into clusters II and III. Cluster II was mixed and contained patients responding or not to AZA while cluster III included only non-responder patients.

To better understand the differences between responders and non-responders, we studied the probes that were located in the promoter. We observed that 61 probes were inside a gene promoter and 58/61 probes were more methylated in responders, whereas only 3/61 probes were less methylated in responder patients. Although the differences between most probes were not significant enough (the average methylation between two groups ranged from 0.009 to 0.226), we focused on those with differences greater than 0.2. These probes affected 4 genes *CRADD*, *RDH13*, *BRDT* and *PACRG*. Only the probe in the *PACRG* gene promoter was hypermethylated (mean methylation = 0.759) in responders, being the β-value methylation nearly to 0.5 in non-responders. The three other genes (*CRADD*, *RDH13* and *BRDT*) showed a *β*-value methylation in responders nearly to 0.47 and hypomethylation in non-responder patients (mean methylation = 0.2666). It is important to note that the *RDH13* gene had five probes in the promoter region, and all were less methylated in non-responder (mean methylation = 0.3644) than in AZA-responder patients (mean methylation = 0.5564).

In conclusion, DNA methylation of a panel of specific sites allows distinguishing responders from non-responders and is possibly related to biological relevant gene regulation.

### A methylation signature at diagnosis predicts longer survival

**Fig. 3:**
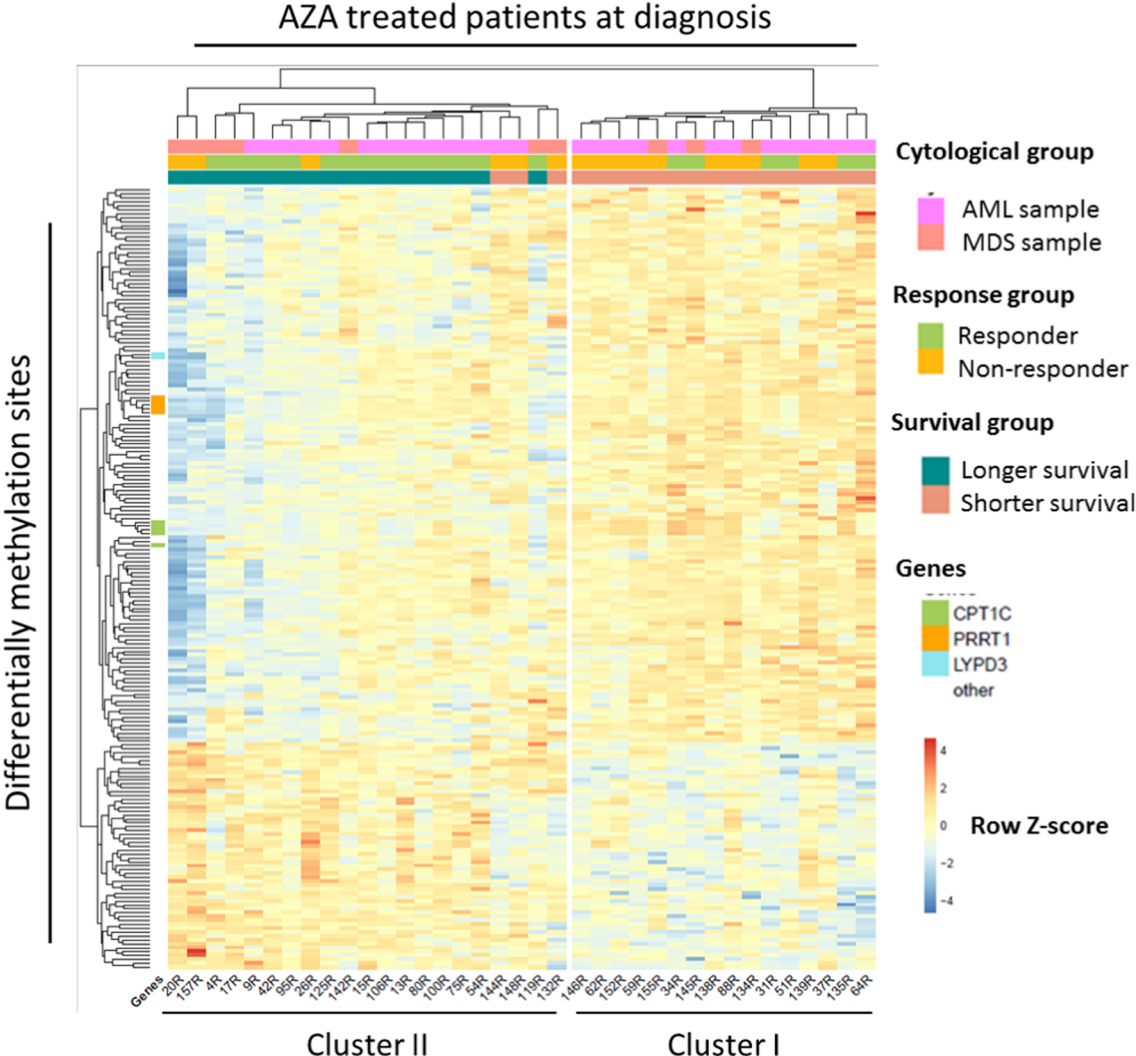
Methylation profile of 200 probes at diagnosis predict overall survival in AZA treated patients. Heatmap showing the hierarchical clusterization of the 200 probes obtained with combined rank analysis that are differentially methylated between longer and shorter survivals in bone marrow samples at diagnosis. Selected genes associated with differentially methylated CpGs are indicated

In order to identify a potential biomarker for OS, we focused on the 37 patients with sample available that received AZA. There were 19 patients who did not reach the median OS (shorter survival) and 18 that exceed the median OS (longer survival). Comparing the two groups with the combined rank analysis, we obtained a methylation signature defined by 200 probes that allowed the differentiation between both groups. When we performed hierarchical clustering with the values of these 200 probes, we classified patients into two major clusters (Fig. 3). Cluster I included exclusively shorter survival patients and cluster II was mixed and contained all patients with longer survival plus 3 patients with shorter survival. We also observed that the majority of patients with longer survival responded to treatment. To better understand the differences between longer and shorter survival groups, we studied the probes that were located in promoter zones. We observed that 73 probes were inside a gene promoter, and there were 3 genes (*CPT1C*, *PRRT1* and *LYPD3*) that had more than two probes differentially methylated between the two groups. All probes were more methylated in shorter survival patients (Additional file 1: Figure 3).

In conclusion, the methylation state of specific CpG sites at diagnosis allows identifying those patients with longer survival.

### Global methylation studies in follow-up samples did not allow predicting which patients would relapse or progress

Using the follow-up samples available, we wanted to see if the methylation profile described at diagnosis that distinguished responding from non-responding patients was able to differentiate patients who would relapse or progress during follow-up. Our cohort included 50 follow-up samples of AZA-treated patients. Of these, 26 samples were from responder patients, 21 were from patients non-responders (12 in disease progression) and 3 samples had no clinical information related to response status.

Hierarchical clustering analysis showed strong clusterization based on patient identity (that means, sequential sample from the same patient were more likely to cluster together) rather than cytological group or treatment category. We observed a decrease in global methylation in the follow-up samples after AZA treatment. This genome-wide demethylation observed after treatment was seen both in responding and non-responding patients, but it was not observed in patients who received ICE without any demethylating agent, demonstrating the demethylation capacity of AZA treatment (data not shown).

**Fig. 4:**
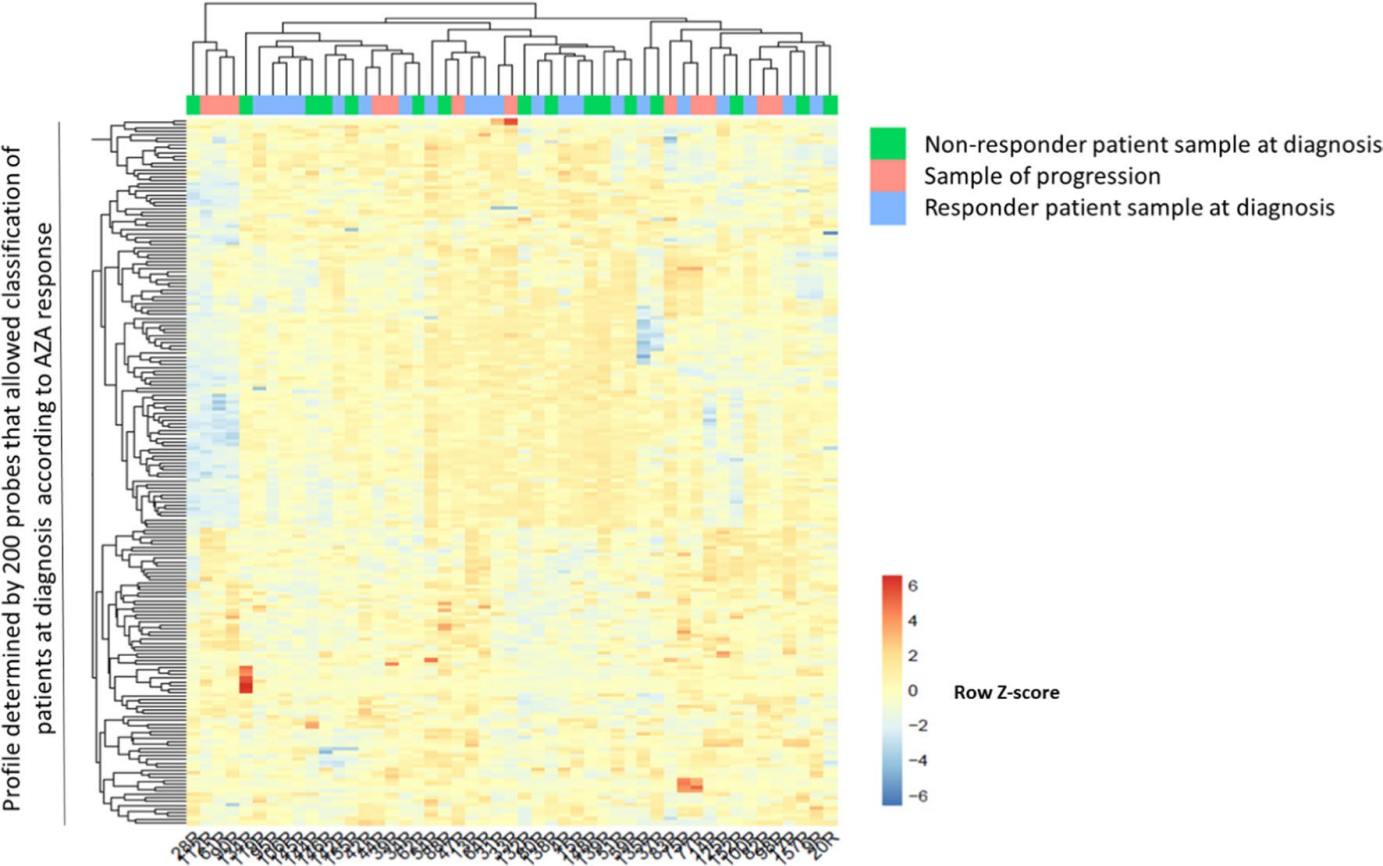
Methylation pattern at time of disease progression after initial response is not similar to methylation pattern from those patients that have never respond (non-responders). Heatmap showing the hierarchical clusterization of samples from patients in progression (*n* =12) in comparison with responder (*n* =21) and non-responder (*n* =16) patients at diagnosis based on DNA methylation of the sites identified in Fig. 2

First, we wished to test if at that time of disease progression after initial response the methylation pattern would be similar to that of non-responders at diagnosis. For this purpose we used the same 200 CpG probes from Fig. 2 to compare samples at disease progression (*n* =12) with diagnostic samples from non-responders (*n* =16) and initial responders (*n* =21). As shown in Fig. 4, we were unable to observe any clear pattern, suggesting that initial and acquired resistances have diverse mechanisms reflected in diverse methylation patterns.

**Fig. 5:**
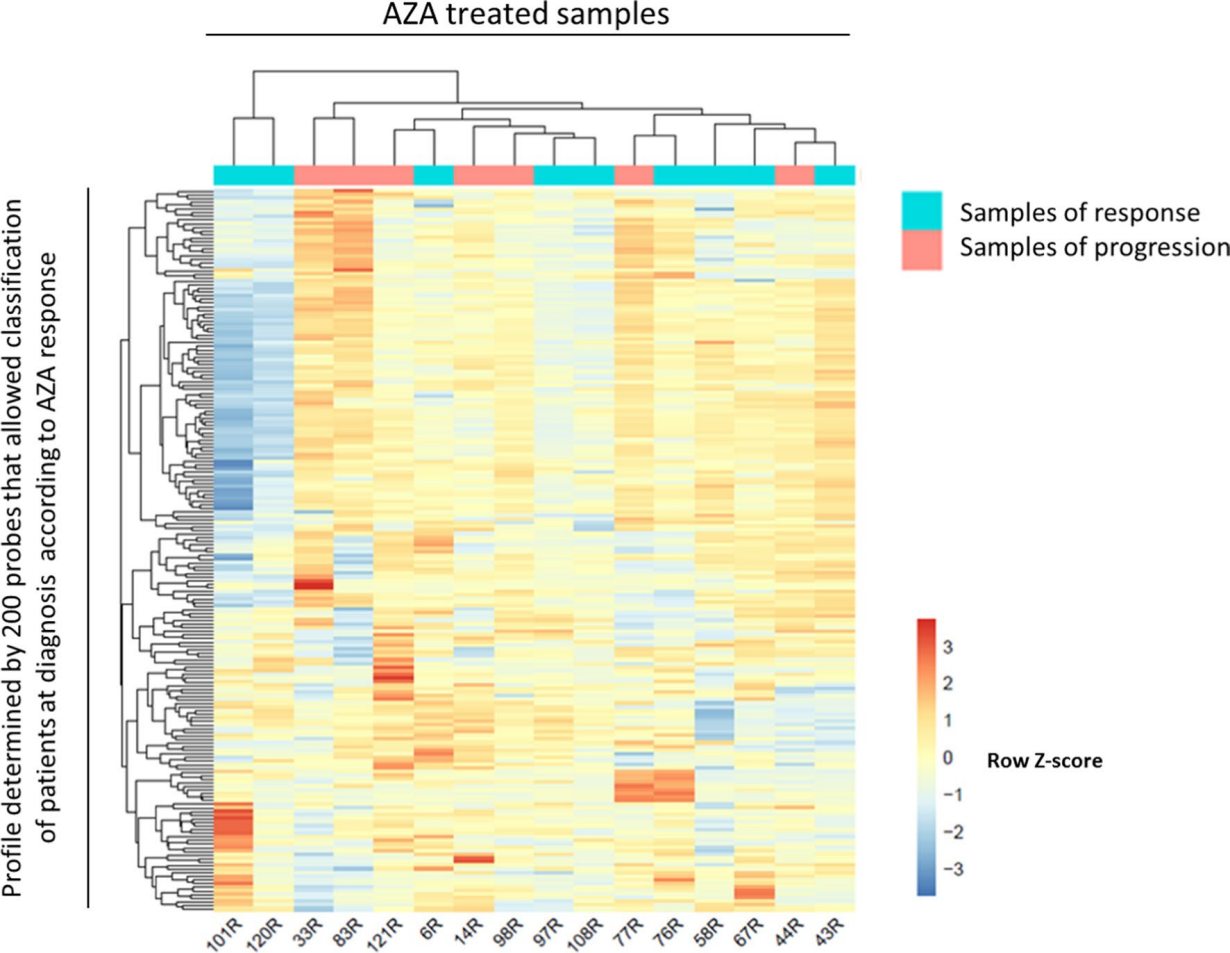
Differential methylation associated with initial response does not provide information on response duration or progression. Heatmap showing the hierarchical clusterization of samples of response after more than 6 AZA cycles (*n* =9) and samples of progression (*n* =7) using the methylation status of CpG sites identified in Fig. 2

Second, we compared the differences between patients that remained in response after six or more cycles of AZA (*n* =9) and patients who, having responded at some point, had progressed to AML (*n* =7) but all of them had received at least 6 AZA cycles (Fig. 5). Again using the same 200 CpG probes, we were unable to distinguish continuous responders from progressors.

In summary, we identified a methylation profile that allowed distinguishing responder and non-responder patients at diagnosis, but the same methylation sites were not informative of duration of response or disease progression at follow-up.

## Discussion

Aberrant DNA methylation plays an essential role in hematopoietic malignancies such as MDS and AML. It has been described that MDS blasts have high rates of mutations in epigenetic modifiers and exhibit altered DNA methylation patterns [39]. Promoter methylation has shown to have a significant role in the pathogenesis and progression of MDS, but a reliable methylation marker, predictive of treatment response has not yet been identified. In this study, we wanted to better understand the DNA methylation pattern at diagnosis and follow-up in patients with high-risk MDS and sAML. In particular, we wished to determine if DNA methylation would allow predicting initial response to AZA at diagnosis and the durability of treatment response and progression.

The effectiveness of HMA in myeloid malignancies could be associated with the fact that hundreds of genes are often hypermethylated in MDS, and that the level of hypermethylation across the genome is linked to poor prognosis and a high likelihood of MDS to transform to AML as well as AML relapse [28, 36, 40, 41]. In our cohort, we observed that high-risk MDS and sAML patients had a different methylation pattern at diagnosis compared to healthy controls, but we found no differences in global genome-wide methylation at diagnosis according to cytological group (MDS versus sAML), cytogenetic groups, type of response to treatment or OS. However, the difference between controls and patients at diagnosis could be influenced by age differences between both groups.

Some years ago Shen et al. [36] described that the decrease in methylation during treatment, rather than methylation at the baseline level, might predict the outcomes of HMA therapy. Similarly, Follo et al. [37] demonstrated that not only was the PI-PLCbeta1 promoter hypermethylated in high-risk MDS patients, but also the amount of PI-PLCbeta1 mRNA during treatment could predict the clinical response to AZA. Conversely, when we performed a combined rank analysis, we observed a specific methylation pattern at diagnosis, defined by 200 probes that could predict which patients would respond to AZA but we did not find any differences in a decrease of methylation during treatment between responding and non-responding patients. In the same way, Meldi et al. [33] identified 167 differentially methylated regions of DNA at baseline in chronic myelomonocytic leukemia that distinguished responders from nonresponders to decitabine, and Voso et al. [34] described that *BCL2L10* methylation at diagnosis may predict response to AZA in MDS patients.

From our study at diagnosis we observed that four genes (*CRADD*, *RDH13*, *BRDT* and *PACRG*) were differentially methylated between responding and non-responding patients to AZA treatment making it of interest to study these genes more in depth. There is scarce information about these genes associated to high-risk MDS or sAML, but *RDH13* seems to be important since it was the only gene with which five probes methylated differently between the two groups. This gene encodes a mitochondrial short-chain dehydrogenase/reductase, which catalyzes the reduction and oxidation of retinoids. The encoded enzyme may function in retinoic acid production and may also protect the mitochondria against oxidative stress. Retinol (vitamin A) is strictly provided by nutrition, and the active retinol product is retinoic acid (RA) which plays a great role as a modulator of proliferation and differentiation in numerous tissues [42]. Retinoic acid receptors (RARs) mediate transcription of different sets of genes controlling differentiation of a variety of cell types, thus the target genes regulated depend upon the target cells [43]. *RDH13* exhibits a wide tissue distribution and, by contrast with other members of the RDH11-like group of short-chain dehydrogenases⁄reductases, it is a mitochondrial rather than a microsomal protein. This protein has a greater catalytic efficiency in the reductive than in the oxidative direction [44]. In our study we found that patients not responding to AZA showed hypomethylation in the *RDH13* promoter, and therefore, we speculate that *RDH13* overexpression might cause deregulation of specific genes managed by RA, involving a change in the expression of other genes that could be related to the lack of response to AZA treatment, but as far as we know, there is no data in the literature that supports our hypothesis. At the same time, the *PACRG* gene also seemed interesting, as its promoter was hypermethylated in responding patients, while this gene promoter in non-responding patients showed a methylation *β*-value next to 0.5. The *PACRG* gene encodes a protein that forms a large molecular complex with chaperones. It has recently been described that the PACRG protein plays an important role in tumor necrosis factor (TNF) signaling, and this function of *PACRG* in positively regulating TNF signaling may help to explain the association of *PACRG* polymorphisms with increased susceptibility to intracellular pathogens [45]. Agirre et al. [46] studied the role of promoter hypermethylation in the regulation of *PACRG* expression in different tumour cell lines and primary patient samples and demonstrated that abnormal methylation resulted in downregulation of *PACRG* gene expression. Although, they focused on different diseases, we can hypothesize that the degree of hypermethylation in responding patients may be explained by the decrease in *PACRG* gene expression, that could be reverted with AZA treatment.

This means that two of the genes detected with a differential methylation signature between responding and non-responding patients to AZA, have a protective function; *RDH13* against oxidative stress and *PACRG* against infections. This suggests that the ability of the cell to cope with stress could be related to the ability of treatment response. Despite the limited size of our cohort and the need for an independent validation cohort, we believe that this profile could be a good tool for future targeted methylation studies.

In 2010, Shen et al. [36] analyzed 317 samples of MDS patients and concluded that DNA methylation of some specific genes predicts OS and PFS in MDS. To be more precise, patients with high methylation levels in ten genes (*CFH1*, *CDH13*, *ERα*, *NOR1*, *NPM2*, *OLIG2*, *p15*, *PGRA*, *PGRB*, *RIL*) had shorter OS and worse PFS. In line with this article, our study defined a panel of 200 probes that correlated a differential methylation pattern at diagnosis according to patients’ survival. Within these probes, there were three genes, *CPT1C*, *PRRT1* and *LYPD3*, of special interest. Patients with shorter survival had their promoters more methylated than patients with longer survival. To our knowledge, the impact of methylation in these genes in OS has not been reported in the literature, but other findings have been described. *CPT1C* is expressed predominantly in mammalian brain and Casals et al. [47] described that *CPT1C* is highly expressed in certain virulent tumor cells, conferring them resistance to glucose-and oxygen-deprivation and therefore, *CPT1C* may be a promising target in the treatment of cancer. *PRRT1* is a 2-pass transmembrane protein belonging to the CD225/Dispanin family and is a component of the outer of AMPAR complex (ionotropic transmembrane receptor for glutamate that mediates fast synaptic transmission in the central nervous system). The proteins of the inner and outer core serve as a platform for other, more peripherally associated AMPAR constituents. Alone or in combination, these auxiliary subunits control the gating and pharmacology of the AMPAR complex and profoundly impact their biogenesis and protein processing [48]. Zhang C et al. [49] describes in their article *PRRT1* as a biomarker of high risk group in breast cancer according to their DNA methylation correlation network. *LYPD3* is a protein that supports cell migration and may be involved in tumor progression [50]. Gruet et al. [51] described that *LYPD3* mRNA expression levels were low in normal tissues, but there is a significant (*P* < 0.001) over-expression of *LYPD3* in several malignant tissues (breast cancer, cervical squamous cell carcinoma and endocervical adenocarcinoma, lung adenocarcinoma, lung squamous cell carcinoma, pancreatic adenocarcinoma, testicular germ cell tumors and thymoma). This suggests that *LYPD3* could be a potential therapeutic target in multiple different cancers. Although all the information found in literature about these genes is referred to solid tumors they could also be of interest for MDS and sAML.

It has also been described that methylation changes in specific genes contribute to disease pathogenesis and may be useful as a marker to monitor the treatment efficacy [52]. Regarding changes in global methylation during treatment, we observed that there is a strong clustering based on patient identity rather than treatment response, as we observed that demethylation occurs in both, responding and non-responding patients. Furthermore, the methylation profile at diagnosis which helped to distinguish responding from non-responding patients was not useful in treated samples (at follow-up). Thus, our hypothesis that the methylation profile at relapse or progression returns to stages similar to those observed at diagnosis in non-responding patients, was not demonstrated, suggesting that the mechanisms that affect relapse/progression of the disease are different from those that predict response at diagnosis.

Aberrant DNA methylation patterns that lead to transcriptional silencing have been recognized as a key epigenetic mechanism in the process of malignant transformation. Although our results are in line with other studies reported in the literature regarding the importance of studying aberrant DNA methylation in MDS and sAML patients, a validation of our findings in longer cohorts would be of interest. In parallel, it would be interesting to assess the functional contribution of differentially methylated genes to provide novel targets for combinatorial therapies to enhance AZA response and to improve OS.

## Conclusions

In summary, in this study we defined a methylation signature at diagnosis that allows the identification of responding and not responding patients to AZA treatment and also another methylation profile that identifies patients with longer survival. This is an important step towards the development of an urgently needed predictive tool for AZA response.

## Methods

### Patients

Bone marrow aspirates from 75 patients with high-risk MDS and sAML were obtained at baseline and during follow-up from 2009 to 2016 (Additional file 1: Table 1). All samples were collected as part of the SMD-09 protocol from the CETLAM Group, a prospective multicenter study. The study was approved by the Institut Català d’Oncologia - Hospital Germans Trias i Pujol Ethics Committee and the Scientific Committee of the CETLAM group. Informed consent was obtained from each patient in accordance with the Declaration of Helsinki. Ten healthy hematopoietic stem cell related donors were also included as normal controls for DNA methylation profiles.

In this protocol patients were classified according to age and comorbidities to determine whether or not they were candidates for intensive treatment. Patients fit for intensive treatment were also stratified according to cytogenetic (Additional file 1: Figure 4). Treatments in the CETLAM SMD-09 protocol included AZA, intensive chemotherapy (idarubicin, cytarabine and etoposide, ICE) and/or allogeneic stem cell transplantation (ASCT) (Additional file 1: Figure 4). We calculated the IPSS and IPSS-R values only for MDS patients, as these prognostic models are specific for this type of patients.

### Response criteria

For response criteria we used the definitions established by the 2006 International Working Group (IWG) for MDS patients [38]. Briefly, patients who achieved complete remission, bone marrow complete remission, partial remission or hematological improvement were considered to be on response. The remaining situations were considered as no response. Response to AZA was assessed, when possible, after cycle 3, 6, 9 and 12 and response to chemotherapy was assessed after cycle 1 or 2.

### Cytogenetic

Conventional G-banding cytogenetic (CC) was performed in bone marrow samples at diagnosis following standard procedures. Karyotypes were described according to the International System for Human Cytogenetic Nomenclature [53].

### DNA samples

Whole bone marrow samples were collected at diagnosis (*n* =73) and at follow-up (*n* =83). Genomic DNA was extracted from the 156 samples (75 patients) and from 10 samples from healthy donors using the QIAamp DNA Blood Mini Kit (Qiagen, Werfen). One microgram of DNA was modified with sodium bisulfate using the EZ DNA methylation kit (Zymo Research, Ecogen) according to the manufacturer’s instructions.

### Methylation array and statistical analysis

Bisulfite converted DNA was then processed and hybridized to the Infinium Human Methylation 450 K BeadChip from Illumina, according to the manufacturer’s recommendations. The 450 K DNA Methylation array assesses the methylation status of 485,764 cytosines distributed over the whole genome, which correspond to CpG dinucleotides (99.3%) and CNG targets (0.7%) [54]. According to their associated RNA transcripts, 361,766 CpGs (74.4%) correspond to classic coding messenger RNA genes, 4,168 (0.85%) are linked to non-coding RNAs (microRNAs and long non-coding RNAs), and for 119,830 (24.6%) sites there are no annotated transcripts associated with the described CpG location [54]. Fluorescence signals generated for unmethylated and methylated cytosines were transformed into a beta-value (β-value) ratio ranging from 0 (completely unmethylated site) to 1 (completely methylated site). Methylation data are available at the Gene Expression Omnibus (GEO) database under accession number GSE152710. Raw IDAT files from Infinium 450 K experiments were imported into RnBeads for DNA methylation analysis, including quality control, data preprocessing and normalization [55]. The methylation β-values were normalized using the BMIQ normalization method [56]. RnBead was used to perform unsupervised principal component analysis (PCA) and to explore associations among traits [55]. Differential methylation analysis was conducted at a site and region level according to the specified sample groups. *P* values of the site level were computed using the limma package [57]. Differential methylation at the site level was computed based on a variety of metrics: (a) comparison of the difference in mean methylation levels of the two groups; (b) the quotient in mean methylation; and c) a statistical test (limma or *t* test) assessing whether the methylation values in the two groups originate from distinct distributions. Additionally, each site was assigned a rank based on each of these 3 criteria. A combined rank is computed as the maximum (i.e. worst) rank among the three ranks. The smaller the combined rank of a site, the more evidence for differential methylation it exhibits. Differential methylation analysis was performed to identify hypermethylated and hypomethylated probes between the compared groups and was performed according to the combined ranking. We analyzed the combined rank among the 200 best ranking sites. Data shown in map are *β*-value *Z*-score converted.

DNA methylation data were assessed and statistically treated as CpG units (consisting of one or more CpG dinucleotides). Treating the region as a unit in the differential methylation analysis allows identifying regions with consistently coordinated methylation changes. Hierarchical clustering was performed using the hclust function, with default parameters from the Bioconductor pheatmap package version 1.0.12. All statistical analyses were performed using RnBeads package version 1.8.0. Post-statistical analyses were done using in-house R scripts.

### Overall Survival and Progression free survival statistical analysis

Overall survival (OS) was defined as time from diagnosis to the last follow-up or death from any cause and progression free survival (PFS) as time from diagnosis to progression or death related to disease. Survival curves were performed using the Kaplan–Meier method and log-rank test was used for comparisons between groups. The statistical package SPSS, version 24.0 (SPSS Inc., Chicago, IL, USA) was used for all analyses.

## Supplementary Information

**Additional file 1**. Supplementary information.

## Abbreviations

AML: Acute myeloid leukemia
ASCT: Allogeneic stem cell transplantation
AZA: Azacitidine
CC: Conventional G-banding cytogenetic
CETLAM: Cooperative group for the study and treatment of acute leukemia and myelodysplastic
DNMT: DNA methyltransferases
GEO: Gene Expression Omnibus
GO: Gene Ontology
HMA: Hypomethylating agents
ICE: Idarubicin, cytarabine and etoposide
IWG: International Working Group
MDS: Myelodysplastic syndromes
PCA: Principal component analysis
RA: Retinoic acid
RARs: Retinoic acid receptors
sAML: Secondary acute myeloid leukemia
