# Supplementary material for: Different methylation signatures at diagnosis in patients with high-risk myelodysplastic syndromes and secondary acute myeloid leukemia predict azacitidine response and longer survival
Source: Clin Epigenetics. 2021 Jan 14;13:9. doi: 10.1186/s13148-021-01002-y (PMC7809812; doi:10.1186/s13148-021-01002-y)
Supplement: Supplementary file 1 — Additional file 1. Supplementary information. [file 13148_2021_1002_MOESM1_ESM.docx]

**Supplementary material**

**Supplementary Figure 1.** Overall Survival and Progression Free Survival of the entire cohort classified according to treatment received.

**Supplementary Figure 2**. Pathways affected by the 109 genes that had the promoter zone more methylated in patients than in controls.

**Supplementary Figure 3.** Differently methylated probes located in promoters that distinguish longer and shorter survival patients.

**Supplementary Figure 4.** Treatment schedule for patients included in the protocol

**Supplementary Table 1**. Classification of samples according to disease time and treatment type.

**Supplementary Table 2**. Classification of patients at diagnosis according to treatment response.

**Supplementary Table 3**. Description of all 156 samples analyzed by Infinium Human Methylation 450K BeadChip from Illumina in this study.

**Supplementary Figure 1.** Overall Survival and Progression Free Survival of the entire cohort classified according to treatment received.


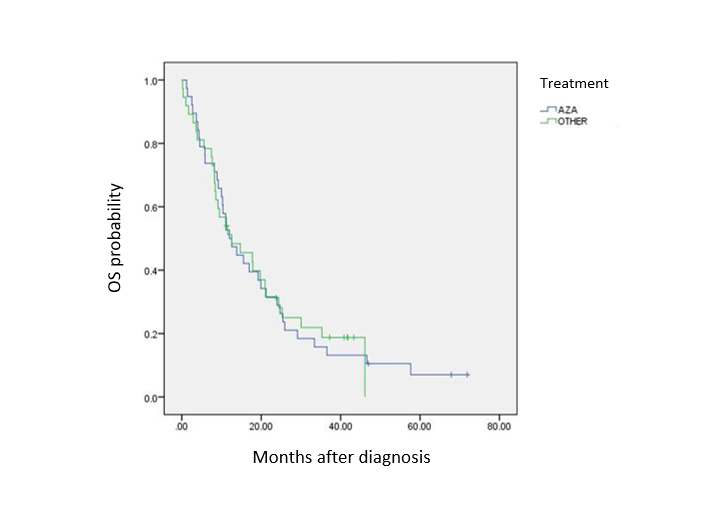

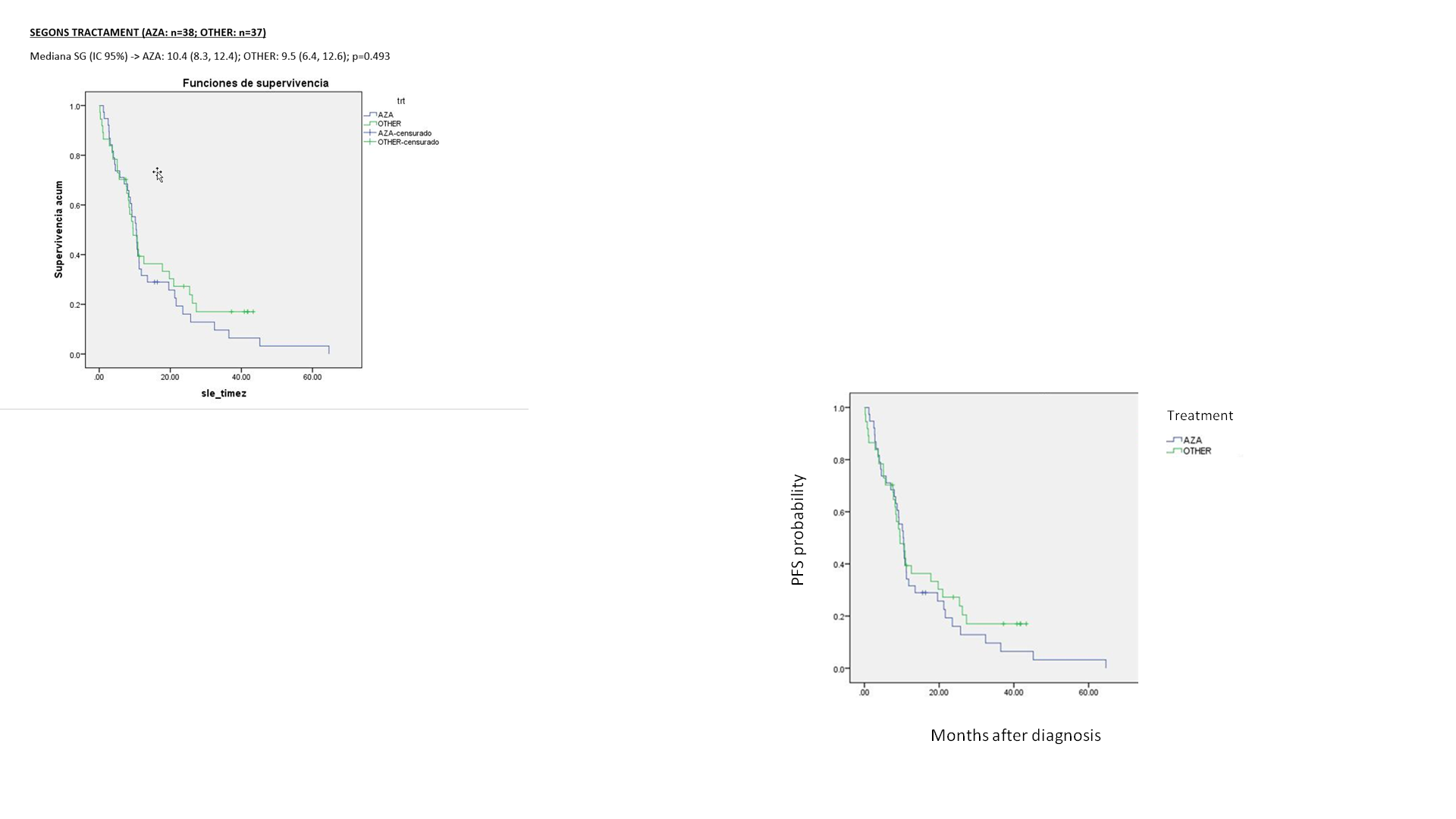


**Supplementary Figure 2**. Pathways affected by the 109 genes that had the promoter zone more methylated in patients than in controls.


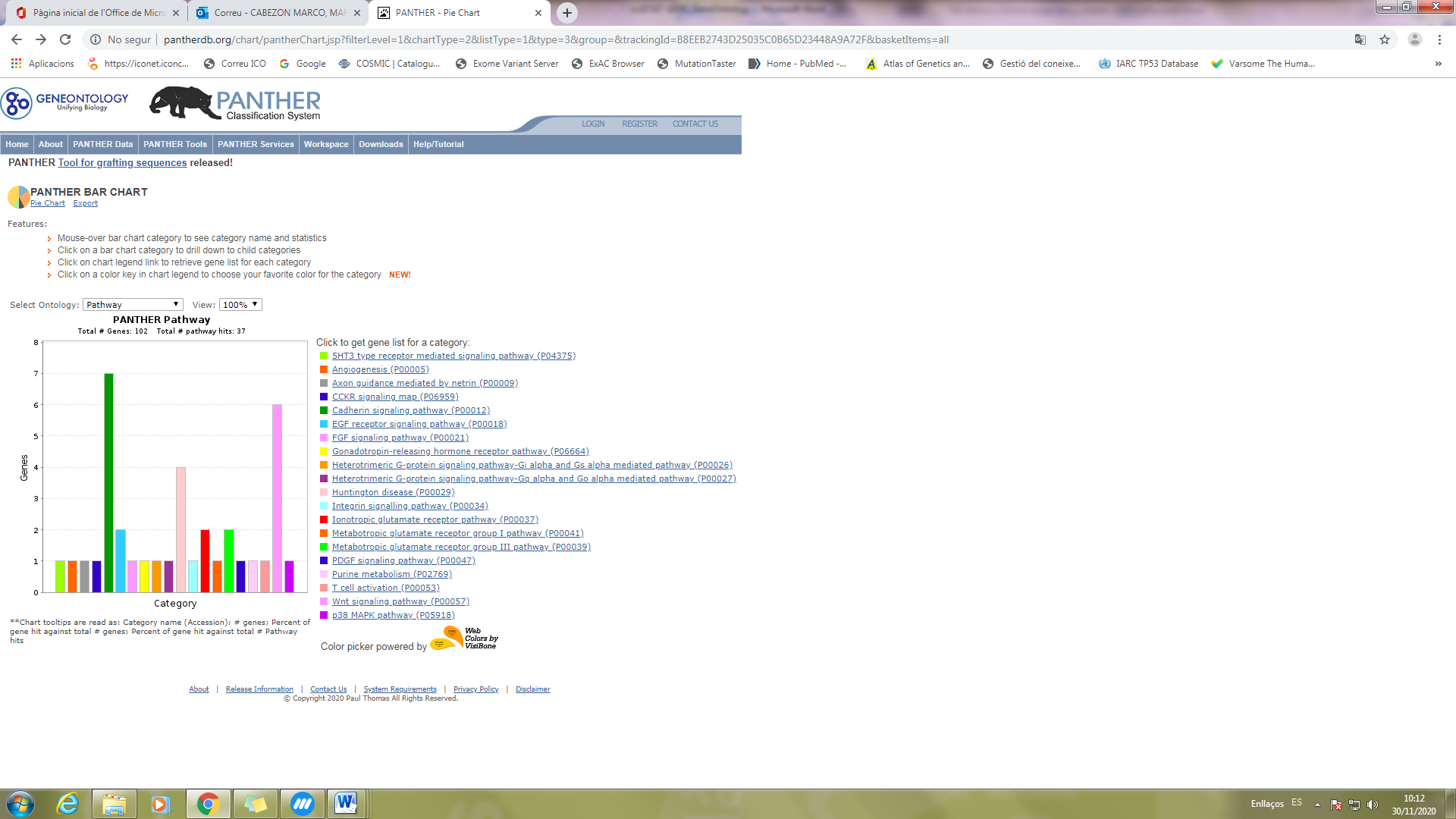


**Supplementary Figure 3.** Differently methylated probes located in promoters that distinguish longer and shorter survival patients.


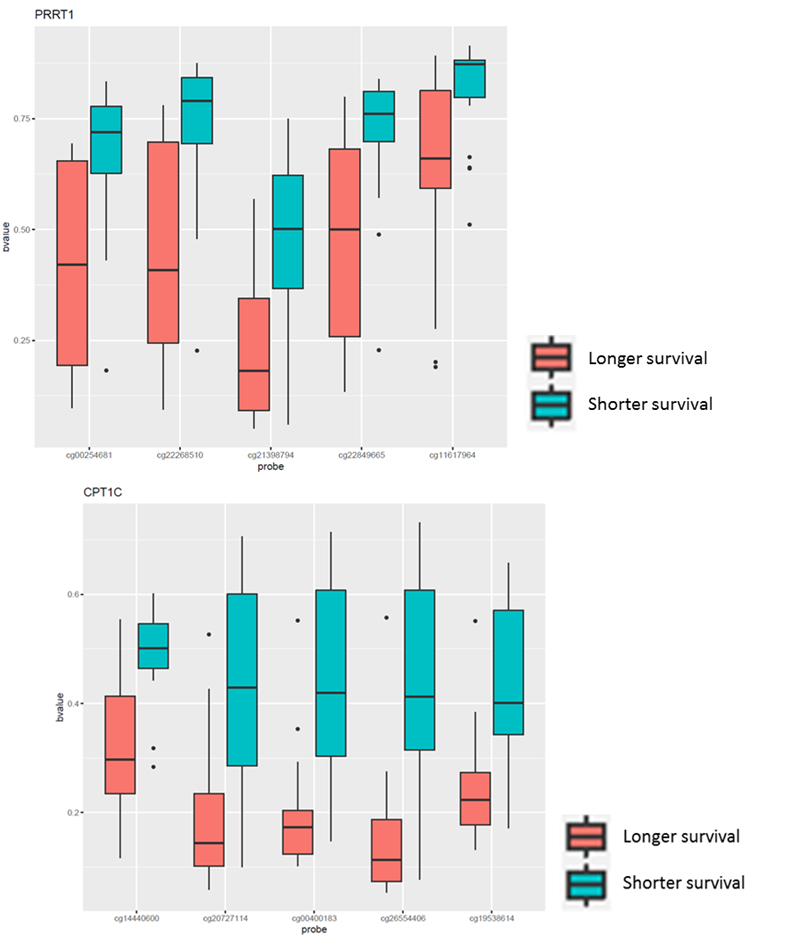


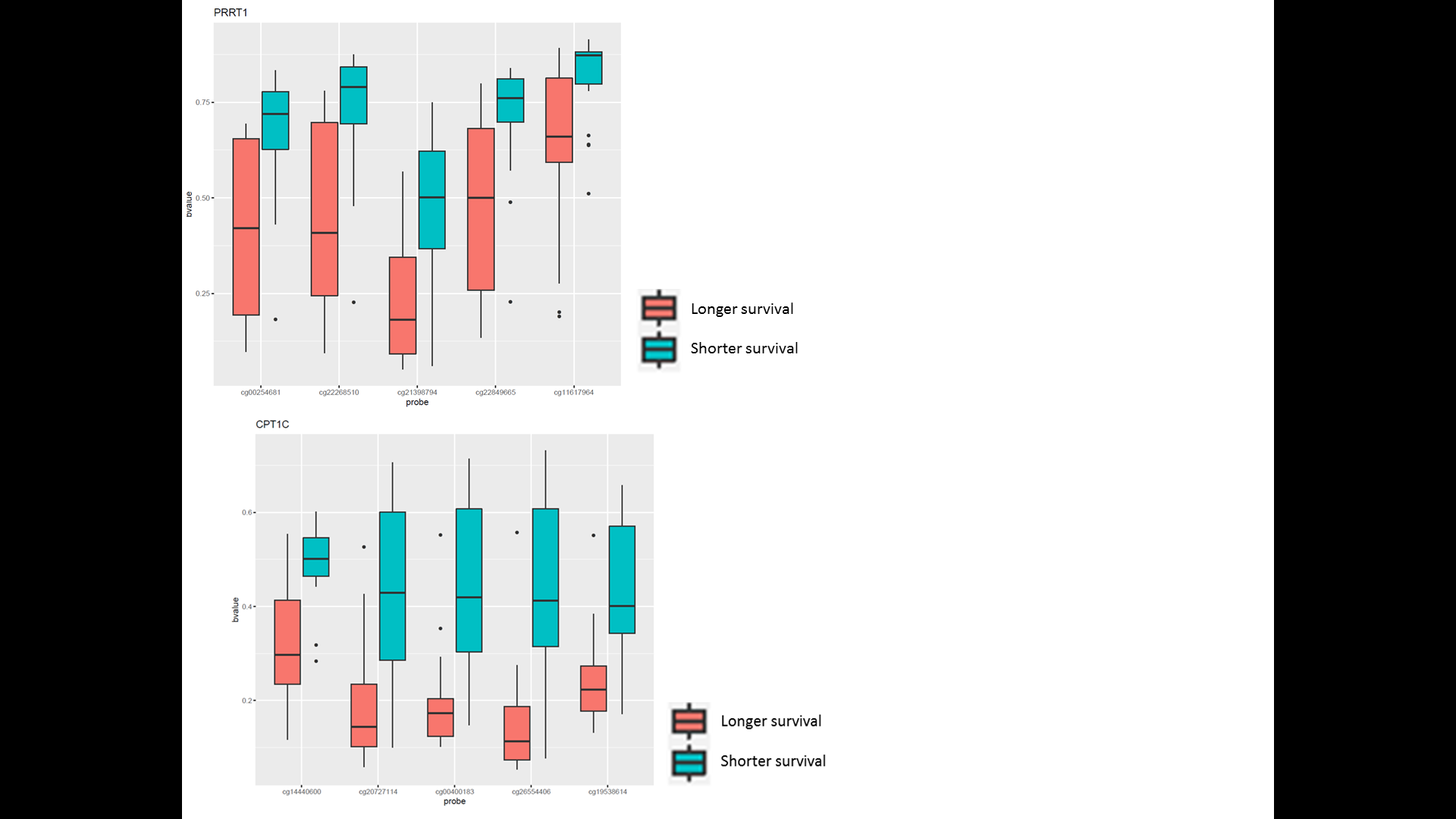


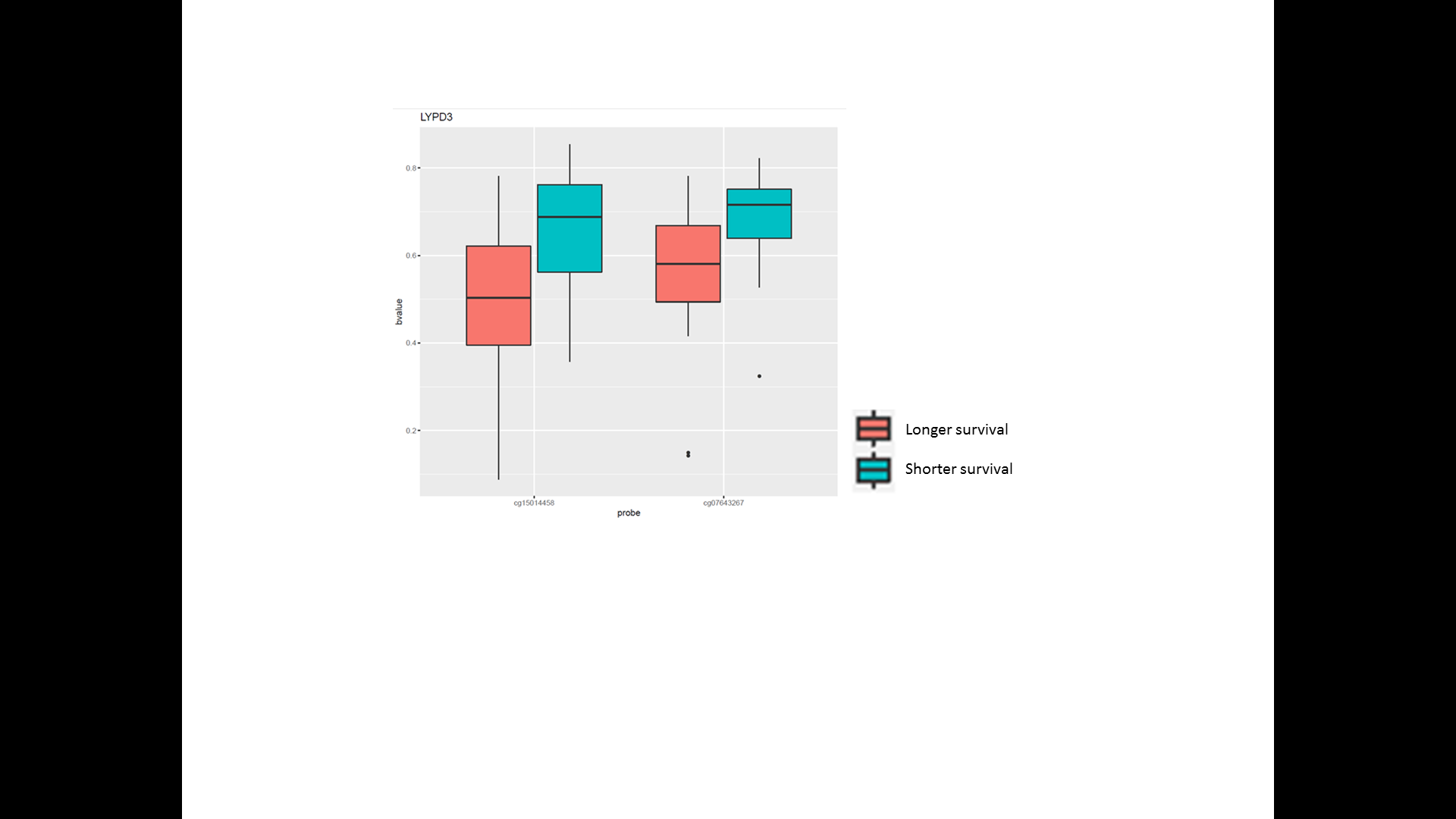


**Supplementary Figure 4.** Treatment schedule for patients included in the protocol


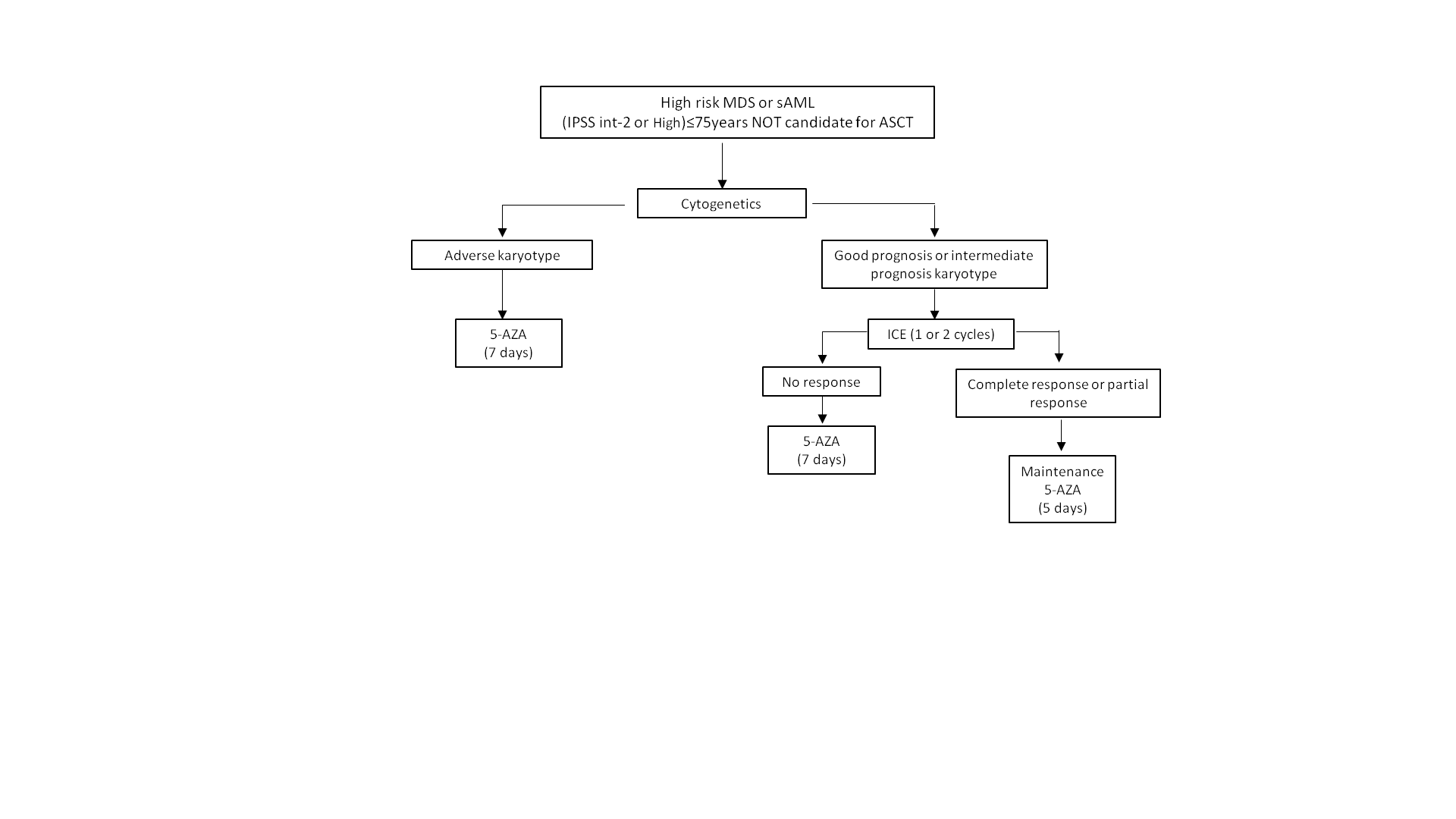


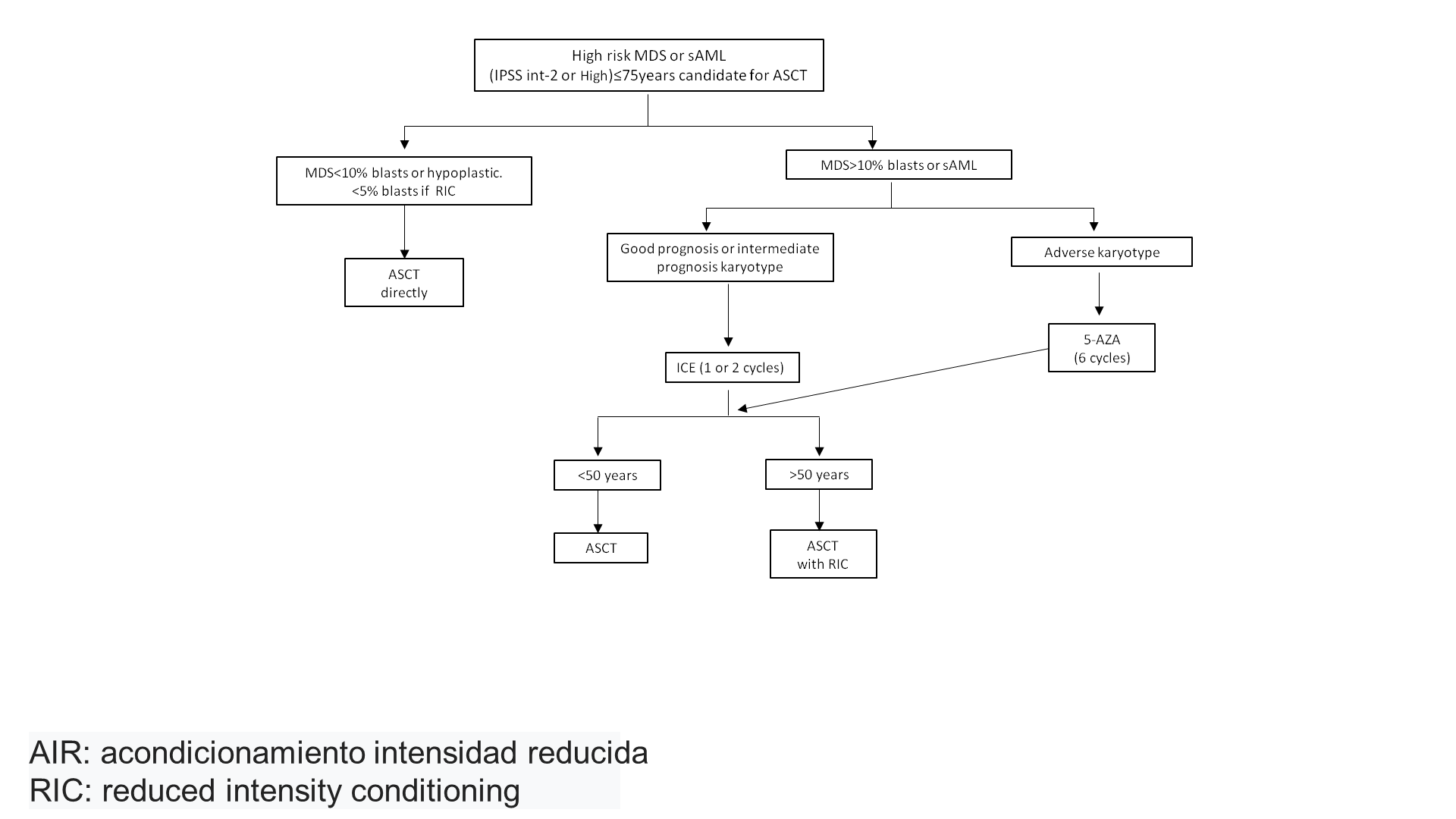


MDS: myelodysplastic syndrome; sAML: secondary acute myeloid leukemia; ASCT: allogeneic stem cell transplantation; AZA: azacitidine; ICE: idarubicin, cytarabine and etoposide; RIC: reduced intensity conditioning.

**Supplementary Table 1**. Classification of samples according to disease time and treatment type.

|  |  | AZA Treatment | Other treatments: chemotherapy (ICE) and/or allogeneic stem cell transplant |
| --- | --- | --- | --- |
| MDS  (49 patients*)  (108 samples) | Diagnosis | 27 | 21 |
|  | Follow-up | 37 | 23 |
| sAML  (26 patients*)  (48 samples) | Diagnosis | 10 | 15 |
|  | Follow-up | 13 | 10 |

MDS: myelodysplastic syndrome; sAML: secondary acute myeloid leukemia; AZA: azacitidine; ICE: idarubicin, cytarabine and etoposide.

*Two patients (one MDS and one sAML) we did not have diagnosis sample but at follow-up.

**Supplementary Table 2**. Classification of patients at diagnosis according to treatment response.

|  | **Samples at diagnosis** | |
| --- | --- | --- |
|  | Responders | Non-responders |
| **AZA treatment (n=37)** | 21 | 16 |
| **Other treatment (n=36)*** | 21 | 10 |

AZA: azacitidine

*There were 5 patients that received other treatment and for whom we did not have response data.

**Supplementary Table 3**. Description of all 156 samples analyzed by Infinium Human Methylation 450K BeadChip from Illumina in this study.

| **Nº patient** | **Sample Nº_ Methylation** | **Disease time point (diagnosis or months from diagnosis)** | **Cytological category group** | **Treatment** | **Sample stage** |
| --- | --- | --- | --- | --- | --- |
| **2** | 1R | Diagnosis | MDS | other | Diagnosis |
| **2** | 2R | 1m | MDS | other | Complete Response |
| **2** | 3R | 3m | MDS | other | Complete Response |
| **3** | 129R | Diagnosis | MDS | other | Diagnosis |
| **4** | 130R | Diagnosis | AML | other | Diagnosis |
| **5** | 131R | Diagnosis | MDS | other | Diagnosis |
| **6** | 4R | Diagnosis | AML | AZA | Diagnosis |
| **6** | 5R | 3m | AML | AZA | No response |
| **6** | 6R | 9m | AML | AZA | Response |
| **7** | 7R | Diagnosis | AML | other | Diagnosis |
| **7** | 8R | 1m | AML | other | Complete response |
| **8** | 132R | Diagnosis | AML | AZA | Diagnosis |
| **9** | 133R | Diagnosis | AML | other | Diagnosis |
| **10** | 10R | 3m | MDS | AZA | Partial Response |
| **10** | 9R | Diagnosis | MDS | AZA | Diagnosis |
| **11** | 134R | Diagnosis | AML | AZA | Diagnosis |
| **12** | 11R | Diagnosis | MDS | other | Diagnosis |
| **12** | 12R | 1m | MDS | other | Partial Response |
| **13** | 13R | Diagnosis | MDS | AZA | Diagnosis |
| **13** | 14R | 6m | MDS | AZA | Progression |
| **14** | 15R | Diagnosis | MDS | AZA | Diagnosis |
| **14** | 16R | 6m | MDS | AZA | No response |
| **15** | 17R | Diagnosis | AML | AZA | Diagnosis |
| **15** | 18R | 12m | AML | AZA | Partial Response |
| **15** | 19R | 24m | AML | AZA | Partial Response |
| **16** | 20R | Diagnosis | AML | AZA | Diagnosis |
| **16** | 21R | 6m | AML | AZA | No response |
| **16** | 22R | 12m | AML | AZA | No response |
| **17** | 135R | Diagnosis | MDS | AZA | Diagnosis |
| **18** | 23R | Diagnosis | MDS | other | Diagnosis |
| **18** | 24R | 4m (sample post TPH) | MDS | other | Not applicable |
| **18** | 25R | 9m (sample post TPH) | MDS | other | Not applicable |
| **19** | 136R | Diagnosis | AML | other | Diagnosis |
| **20** | 137R | Diagnosis | MDS | other | Diagnosis |
| **21** | 26R | Diagnosis | MDS | AZA | Diagnosis |
| **21** | 27R | 3m | MDS | AZA | No response |
| **21** | 28R | 6m | MDS | AZA | No response |
| **22** | 29R | Diagnosis | AML | other | Diagnosis |
| **22** | 30R | 1m | AML | other | No response |
| **24** | 138R | Diagnosis | MDS | AZA | Diagnosis |
| **25** | 31R | Diagnosis | MDS | AZA | Diagnosis |
| **25** | 32R | 3m | MDS | AZA | Partial Response |
| **25** | 33R | 6m | MDS | AZA | Progression |
| **26** | 139R | Diagnosis | MDS | AZA | Diagnosis |
| **27** | 140R | 1m | AML | other | No response |
| **29** | 141R | Diagnosis | MDS | other | Diagnosis |
| **30** | 142R | Diagnosis | AML | AZA | Diagnosis |
| **31** | 34R | Diagnosis | MDS | AZA | Diagnosis |
| **31** | 35R | 3m | MDS | AZA | Partial Response |
| **31** | 36R | 9m (sample post TPH) | MDS | other | Not applicable |
| **32** | 37R | Diagnosis | MDS | AZA | Diagnosis |
| **32** | 38R | 2m | MDS | AZA | No response |
| **32** | 39R | 5m | MDS | AZA | Progression |
| **34** | 40R | Diagnosis | AML | other | Diagnosis |
| **34** | 41R | 1m | AML | other | No response |
| **35** | 143R | Diagnosis | AML | other | Diagnosis |
| **37** | 144R | Diagnosis | MDS | AZA | Diagnosis |
| **38** | 42R | Diagnosis | MDS | AZA | Diagnosis |
| **38** | 43R | 24m | MDS | AZA | Partial Response |
| **38** | 44R | 32m | AML | AZA | Progression |
| **38** | 45R | 38m | AML | AZA | No data |
| **38** | 46R | 41m | AML | AZA | No data |
| **38** | 47R | 44m | AML | AZA | Progression |
| **39** | 48R | Diagnosis | AML | other | Diagnosis |
| **39** | 49R | 1m | AML | other | Complete response |
| **39** | 50R | 3m | AML | other | Progression |
| **40** | 145R | Diagnosis | AML | AZA | Diagnosis |
| **41** | 51R | Diagnosis | MDS | AZA | Diagnosis |
| **41** | 52R | 3m | MDS | AZA | Partial Response |
| **41** | 53R | 6m | MDS | AZA | Complete Response |
| **42** | 146R | Diagnosis | MDS | AZA | Diagnosis |
| **43** | 147R | Diagnosis | AML | other | Diagnosis |
| **44** | 54R | Diagnosis | MDS | AZA | Diagnosis |
| **44** | 55R | 3m | MDS | AZA | Partial Response |
| **44** | 56R | 6m | MDS | AZA | Partial Response |
| **44** | 57R | 9m | MDS | AZA | Partial Response |
| **44** | 58R | 12m | MDS | AZA | Partial Response |
| **45** | 59R | Diagnosis | MDS | AZA | Diagnosis |
| **45** | 60R | 3m | MDS | AZA | No response |
| **45** | 61R | 6m | MDS | AZA | Progression |
| **46** | 148R | Diagnosis | MDS | AZA | Diagnosis |
| **47** | 149R | Diagnosis | AML | other | Diagnosis |
| **48** | 62R | Diagnosis | MDS | AZA | Diagnosis |
| **48** | 63R | 3m | MDS | AZA | No response |
| **49** | 64R | Diagnosis | MDS | AZA | Diagnosis |
| **49** | 65R | 3m | MDS | AZA | Partial Response |
| **50** | 66R | 12m | MDS | AZA | Complete Response |
| **50** | 67R | 18m | MDS | AZA | Partial Response |
| **51** | 68R | Diagnosis | MDS | other | Diagnosis |
| **51** | 69R | 1m | MDS | other | Complete Response |
| **51** | 70R | 5m | MDS | other | Complete Response |
| **51** | 71R | 6m | MDS | other | Complete Response |
| **52** | 72R | Diagnosis | AML | other | Diagnosis |
| **52** | 73R | 3m | AML | other | Complete Response |
| **52** | 74R | 7m | AML | other | Progression |
| **53** | 150R | Diagnosis | AML | other | Diagnosis |
| **54** | 75R | Diagnosis | MDS | AZA | Diagnosis |
| **54** | 76R | 6m | MDS | AZA | Complete Response |
| **54** | 77R | 12m | MDS | AZA | Progression |
| **55** | 151R | Diagnosis | MDS | other | Diagnosis |
| **56** | 78R | Diagnosis | MDS | other | Diagnosis |
| **56** | 79R | 1m | MDS | other | No response |
| **57** | 80R | Diagnosis | MDS | AZA | Diagnosis |
| **57** | 81R | 3m | MDS | AZA | Partial Response |
| **57** | 82R | 12m | MDS | AZA | Progression |
| **57** | 83R | 16m | MDS | AZA | Progression |
| **58** | 84R | Diagnosis | MDS | other | Diagnosis |
| **58** | 85R | 3m | MDS | other | No response |
| **59** | 86R | Diagnosis | MDS | other | Diagnosis |
| **59** | 87R | 2m | MDS | other | No data |
| **60** | 88R | Diagnosis | MDS | AZA | Diagnosis |
| **60** | 89R | 3m | MDS | AZA | No response |
| **60** | 90R | 6m | AML | AZA | Progression |
| **61** | 152R | Diagnosis | MDS | AZA | Diagnosis |
| **62** | 93R | Diagnosis | MDS | other | Diagnosis |
| **62** | 94R | Diagnosis | MDS | other |  |
| **63** | 153R | Diagnosis | AML | other | Diagnosis |
| **64** | 95R | Diagnosis | MDS | AZA | No response |
| **64** | 96R | 3m | MDS | AZA | Complete Response |
| **64** | 97R | 6m | MDS | AZA | Partial Response |
| **64** | 98R | 12m | MDS | AZA | Progression |
| **64** | 99R | 15m | MDS | AZA | No data |
| **65** | 100R | Diagnosis | MDS | AZA | Diagnosis |
| **65** | 101R | 12m | MDS | AZA | Complete Response |
| **66** | 154R | Diagnosis | MDS | other | Diagnosis |
| **67** | 102R | Diagnosis | MDS | other | Diagnosis |
| **67** | 103R | 1m | MDS | other | No data |
| **68** | 155R | Diagnosis | AML | AZA | Diagnosis |
| **69** | 104R | Diagnosis | MDS | other | Diagnosis |
| **69** | 105R | 1m | MDS | other | No data |
| **71** | 106R | Diagnosis | MDS | AZA | Diagnosis |
| **71** | 107R | 3m | MDS | AZA | Partial Response |
| **71** | 108R | 6m | MDS | AZA | Partial Response |
| **72** | 156R | Diagnosis | MDS | other | Diagnosis |
| **73** | 109R | Diagnosis | MDS | other | Diagnosis |
| **73** | 110R | 1m | MDS | other | Complete Response |
| **74** | 111R | Diagnosis | MDS | other | Diagnosis |
| **74** | 112R | 7m | MDS | other | Partial Response |
| **74** | 113R | 12m | MDS | other | Partial Response |
| **74** | 114R | 18m | MDS | other | Partial Response |
| **77** | 157R | Diagnosis | AML | AZA | Diagnosis |
| **78** | 115R | Diagnosis | MDS | other | Diagnosis |
| **78** | 116R | 1m | MDS | other | Complete Response |
| **78** | 117R | 3m | MDS | other | Complete Response |
| **78** | 118R | 6m | MDS | other | Complete Response |
| **79** | 158R | Diagnosis | AML | other | Diagnosis |
| **80** | 119R | Diagnosis | AML | AZA | Diagnosis |
| **80** | 120R | 6m | AML | AZA | Complete Response |
| **80** | 121R | 12m | AML | AZA | Progression |
| **81** | 122R | Diagnosis | AML | other | Diagnosis |
| **81** | 123R | 1m | AML | other | Complete Response |
| **81** | 124R | 5m | AML | other | Complete Response |
| **82** | 125R | Diagnosis | MDS | AZA | Diagnosis |
| **82** | 126R | 3m | MDS | AZA | Partial Response |
| **83** | 127R | Diagnosis | MDS | other | Diagnosis |
| **83** | 128R | 3m | MDS | other | No data |

**Complete response**: Bone marrow <5% myeloblasts with normal maturation of all cell lines.

**Partial response**: Bone marrow blasts decreased by ≥ 50% over pre-treatment but still >5% blasts.

**No response**: Bone marrow >5% without a decrease by 50% over pre-treatment.

**Progression**: For patients with:

Less than 5% blasts: ≥ 50% increase in blasts to > 5% blasts

5%-10% blasts: ≥ 50% increase to > 10% blasts

10%-20% blasts: ≥ 50% increase to > 20% blasts

20%-30% blasts: ≥ 50% increase to > 30% blasts
